# Pathways between depression, substance use and multiple sex partners among Northern and Indigenous young women in the Northwest Territories, Canada: results from a cross-sectional survey

**DOI:** 10.1136/sextrans-2017-053265

**Published:** 2017-10-07

**Authors:** Carmen H Logie, Candice Lys, Moses Okumu, Cristina Leone

**Affiliations:** 1 Factor-Inwentash Faculty of Social Work, University of Toronto, Toronto, Ontario, Canada; 2 Women’s College Research Institute, Women’s College Hospital, University of Toronto, Toronto, Ontario, Canada; 3 Dalla Lana School of Public Health, University of Toronto, Toronto, Ontario, Canada; 4 Fostering Open eXpression among Youth (FOXY), Yellowknife, Northwest Territories, Canada; 5 Department of Psychology, University of Toronto, Toronto, Ontario, Canada

**Keywords:** sexually transmitted diseases, adolescent, depression, women, substance-related disorders, American native continental ancestry group

## Abstract

**Objectives:**

Sexual and mental health disparities exist in the Northwest Territories (NWT) compared with other Canadian regions. STI rates are 10-fold higher, and youth suicide rates double the Canadian average. Scant research has examined associations between mental and sexual health among youth in the NWT. The study objective was to explore pathways from depression to multiple sex partners (MSP) among young women in the NWT, Canada.

**Methods:**

We implemented a cross-sectional survey in 2015–2016 with a venue-based recruitment sample of young women aged 13–17 attending secondary schools in 17 NWT communities. We conducted path analysis to test a conceptual model examining associations between depression and a history of MSP, examining substance use and peer support as mediators.

**Results:**

Participants (n=199; mean age: 13.8, SD: 1.27) mostly identified were Indigenous (n=154; 77.4%) and one-fifth (n=39; 20.5%) were sexually diverse/non-heterosexual. Almost two-thirds (n=119; 63.3%) reported depression symptoms. One-quarter (n=53; 26.6%) were currently dating, and 16.1% (n=32) reported a lifetime history of >1 sex partner (classified as having MSP). There was no direct effect between depression and MSP (β=0.189, p=0.087, 95% CI 0.046 to 0.260). Depression had a direct effect on substance use (β=0.023, p<0.050, 95% CI 0.118 to 0.500), and an indirect effect on MSP through substance use (β=0.498, *SE*=0.10, p<0.001, 95% CI 0.141 to 0.280). Depression was associated with lower peer support (β=−0.168, p<0.010, 95% CI −0.126 to 0.280); peer support was not associated with MSP (β=−0.158, p=0.130, 95% CI −0.126 to 0.001).

**Conclusion:**

This research is among the first to identify mental health factors associated with STI vulnerability among young women in the NWT. Findings demonstrate the importance of addressing depression and substance use in sexual health interventions in Northern contexts.

## Introduction

There are profound sexual and mental health disparities in the Northwest Territories (NWT), with youth STI rates 10-fold higher,^1^ and youth suicide rates double^2^ the Canadian average. Depression, substance use and sexual practices,^3^ such as sex with multiple partners, elevate adolescents’ vulnerability to STI acquisition. Despite these Northern youth health disparities, little is known about pathways between depression and sexual risk practices in the NWT.^4^ Adolescent girls are particularly vulnerable to STI due to biological factors, including the developmental stage of the cervical opening and cervical mucus, and social factors such as inequitable gender norms that constrain condom negotiation.^5^

Engaging in sex with multiple partners, a risk factor for STI acquisition and transmission, is associated with myriad factors. Substance use and low self-esteem could independently or collectively contribute to engagement in multiple sexual relationships; having sex with multiple partners could also be a coping mechanism for depression.^3^ Adolescent women with higher levels of peer support have lower depression risks^6^ as well as reduced sexual risk practices.^6^ Pathways between depression and sexual risk practices among Northern young women are underexplored. We examined peer support and substance use as mediators of the association between depression and multiple sex partners (MSP) among young women in the NWT.

## Methods

### Study design

We conducted a self-administered pen-and-paper cross-sectional survey with self-identified women aged 13–17 recruited using venue-based sampling at secondary schools across the NWT. Girls in Grade 7–12 classes at schools in 17 NWT communities (Aklavik, Whati, Fort McPherson, N’Dilo, Lutselk’e, Fort Liard, Fort Simpson, Yellowknife, Ulukhaktok, Fort Resolution, Behchoko, Inuvik, Tuktoyaktuk, Hay River, Katlodeeche First Nation, Fort Smith and Norman Wells) who agreed to participate in sexual health workshops were invited to take part in the voluntary survey before the workshop. Trained research assistants implemented data collection, including describing consent processes. All participants provided written consent prior to completing the survey. This study addressed two research questions: (a) is there a direct relationship between depression and reporting MSP? and (b) do peer support and substance use mediate the relationship between depression and MSP? Research ethics approval was attained from the Research Ethics Board at the University of Toronto (REB #31602), Toronto, Ontario and the Aurora Research Institute, Aurora College, Inuvik, NWT License # 15 741.

### Participants and eligibility

Inclusion criteria for study participants included persons who self-identified as women; were aged 13–17 years old; resided in the NWT and were able to provide written informed consent.

### Survey measures

Participants were asked to report their lifetime number of sex partners with the question: ‘How many people have you had sex with (vaginal or anal) in your life?’ Participants reporting more than one sex partner were classified as having MSP. The Patient Health Questionnaire-2 (PHQ-2) was used to screen for depression^7^ (Cronbach’s alpha=0.62, scale range 0–8). Following recommended guidelines, participants with a score >3 were classified as depressed.^7^ We assessed peer support and substance use as potential mediators. Peer support was assessed using a two-item subscale from the Child & Youth Resilience Measure^8^ (Cronbach’s alpha=0.84, scale range 1–6). Substance use was assessed with a 5-point Likert scale item: ‘how frequently do you use drugs or alcohol?’

### Statistical analyses

We conducted descriptive and bivariate analyses using Statistical Package for the Social Sciences (SPSS) V.23 to explore sample characteristics, and independent t-tests and Χ^2^ analyses to determine sociodemographic differences in outcome, explanatory and mediator variables. Mann-Whitney U tests were conducted to compare median scores across demographic variables due to the non-normal distribution of the substance use variable. We summed scale items and recoded the MSP variable into a dichotomous outcome. We conducted Pearson correlation to test for multicollinearity between continuous independent and mediating variables. Path analysis was conducted in Mplus V.7.4 with means-adjusted and variance-adjusted weighted least squares (WLSMV) estimation method; this method is well suited for ordinal variables. With a binary outcome, the standardised coefficient of the predictors will be associated with having MSP (coded as 1). The bootstrapping method with bias-corrected confidence estimates was used to assess the significance of the indirect path from depression to MSP. The percentage of cases with missing values was <5% among variables in these analyses. We used pairwise deletions as default means of handling missing data for bivariate analysis and full information maximum likelihood for the path analysis.

## Results

### Study population

There were 199 participants (mean age: 13.8, SD: 1.27) with a response rate of 98%; three-quarters were identified as Indigenous (n=154; 77.4%) and one-fifth (n=39; 20.5%) as sexually diverse/non-heterosexual. One-quarter of participants (n=53; 26.6%) were currently in a relationship and 16.1% (n=32) reported a history of MSP. Depression scores ranged from 2 to 8 with a mean of 4.22 (SD=1.59), with most of the participants reporting depression symptoms (n=119; 63.3%). Adolescent women’s peer support ranged from 1 to 5 with a mean of 3.64 (SD=1.23). About a third of the participants (n=67; 33.7%) reported using substances (alcohol, weed, speed, cocaine and ecstasy). Frequency of substance use ranged from 0 to 4 with a mean of 1.17 (SD=1.71). One-quarter of participants (n=40; 20.9%) used substances once a month, 6.8% (n=13) once a week, 4.7% (n=9) most days of the week and 1% (n=2) used substances everyday.

We conducted bivariate analyses of outcomes and sociodemographic factors, Indigenous identity and sexual orientation widely associated with mental and sexual health disparities. Sexual minorities (*M*=4.932, SD=1.75) reported higher depression symptoms than heterosexuals (*M*=4.09, SD=1.49; *t*=−3.02, p<0.05). Indigenous participants reported higher substance use than non-Indigenous participants (*M* rank=98.59 vs 76.58; Mann-Whitney U test, *Z=−2.69, p<0.01*). Neither was correlated with MSP.

### Path analysis results

**Figure 1:**
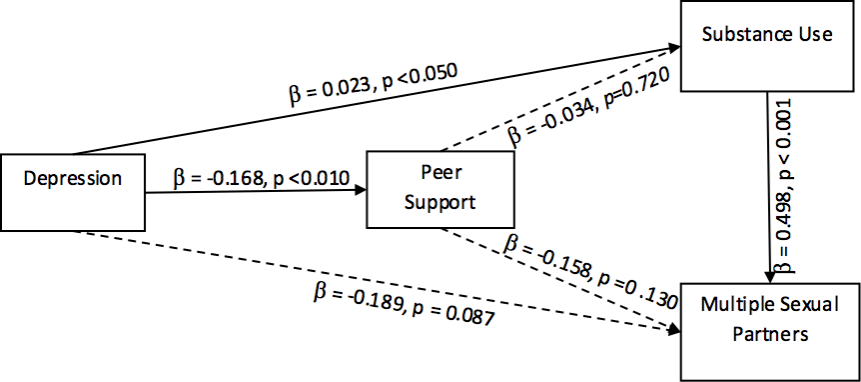
Tested conceptual model for the hypothesized relationships between depression and MSP among Northern and indigenous adolescent girls in the NWT, Canada (n=199). MSP, multiple sex partners; NWT, Northwest Territories.

Results are depicted in figure 1. There was no effect from depression to MSP (β=0.189, p=0.087, 95% CI −0.046 to 0.260). Results indicate that depression has a direct association with substance use (β=0.023, p<0.050, 95% CI 0.118 to 0.500), and a indirect association with MSP through the mediating role of substance use (β=0.498, p<0.001, 95% CI 0.141 to 0.280). Meaning, substance use increases by 0.023 standardised unit for every 1 standardised unit increase in depression. When depression increases by 1 standardised unit, it indirectly leads to a 0.498 increase in the predicated probability of having MSP through the mediator of substance use. The bootstrap test for indirect effects confirms that depression indirectly affects MSP through substance use (ie, indirect effect: β=0.10, p<0.050, 95% CI 0.032 to 0.170).

Depression was negatively associated with peer support (β=−0.168, p<0.010, 95% CI −0.470 to –0.031). There was no direct effect between peer support and substance use (β=−0.034, p=0.720, 95% CI −0.164 to 0.085) or peer support and MSP (β=−0.158, p=0.130, 95% CI −0.126 to 0.001). Thus, peer support was not a significant mediator in the associations between depression, substance use and MSP.

## Discussion

In this study with young women from the NWT, substance use mediated the effect of depression on MSP. Nearly two-thirds (n=119; 63.3%) of participants reported depression symptoms, and one-third (n=67; 33.7%) reported substance use. This study is among the first to explore pathways between depression and sexual practices among young women in the NWT: a highly relevant context to focus on with national sexual and mental health disparities.

Findings corroborate prior research^3^ in other contexts that explore how depression contributes to STI vulnerability. For instance, Shrier *et al* 3 reported that depression was associated with sexual risk practices, and that adolescents women who were depressed often used substances before engaging in sex. The associations we document between mental (depression and substance use) and sexual health suggest the utility of a syndemic approach to research and interventions that considers the synergistic effect of multiple health challenges^9^ on overall well-being among young Northern women.

Similar to other research, we found that depression was associated with lower peer support. In contrast with prior research,^6^ we found peer support was not directly or indirectly associated with MSP. Participants’ peer networks may not have the knowledge and tools to provide insightful information on sexual practices. Although the path coefficients between peer support and substance use or MSP were not significant, the negative direction of these associations are consistent with literature^6^ on the protective role of peer support in youth well-being.

Study limitations include non-random sampling that limits generalisability. The cross-sectional nature precludes inferring causality between variables. We did not collect biological assays to assess STI outcomes; this could strengthen study findings. Future research could also further explore factors associated with engaging in sex under the influence of substances.

Findings can inform targeted intervention development taking into consideration unique needs of young Northern women who are Indigenous and sexual minorities. Indigenous health disparities, such as higher substance use, are rooted in larger sociopolitical and historical contexts of colonisation and intergenerational trauma from residential schools.^10^ Approaches that address syndemics of depression and substance use,^9^ and build knowledgeable peer networks, hold promise in promoting sexual health among young women in Northern and rural communities.

## References

[1] Public Health Agency of Canada. Report on sexually transmitted infections in Canada. 2010 http://www.phac-aspc.gc.ca/sti-its-surv-epi/rep-rap-2012/index-eng.php

[2] Northwest Territories Health and Services. Northwest territories health status report. 2011 http://www.assembly.gov.nt.ca/sites/default/files/11-08-22td60-166.pdf (accessed 22 Jan 2017).

[3] Shrier LA, Harris SK, Sternberg M, et al self-esteem, and substance use with sexual risk among adolescents. Prev Med 2001;33:179–89. 11522159 10.1006/pmed.2001.0869

[4] Lys C, Reading C Coming of age: how young women in the Northwest Territories understand the barriers and facilitators to positive, empowered, and safer sexual health. Int J Circumpolar Health 2012;71:18957 10.3402/ijch.v71i0.18957 PMC3417544 22765935

[5] Wong T, Singh A, Mann J, et al Gender differences in bacterial STIs in Canada. BMC Womens Health 2004;4 Suppl 1:S26 10.1186/1472-6874-4-S1-S26 15345089 PMC2096668

[6] Brady SS, Dolcini MM, Harper GW, et al Supportive friendships moderate the association between stressful life events and sexual risk taking among African American adolescents. Health Psychol 2009;28:238–48. 10.1037/a0013240 19290716 PMC2657930

[7] Kroenke K, Spitzer RL, Williams JB The patient health questionnaire-2: validity of a two-item depression screener. Med Care 2003;41:1284–92. 10.1097/01.MLR.0000093487.78664.3C 14583691

[8] Liebenberg L, Ungar M, Vijver FVde Validation of the child and youth resilience measure-28 (CYRM-28) among Canadian youth. Res Soc Work Pract 2012;22:219–26. 10.1177/1049731511428619

[9] Singer M, Clair S Syndemics and public health: reconceptualizing disease in bio-social context. Med Anthropol Q 2003;17:423–41. 10.1525/maq.2003.17.4.423 14716917

[10] King M, Smith A, Gracey M Indigenous health part 2: the underlying causes of the health gap. Lancet 2009;374:76–85. 10.1016/S0140-6736(09)60827-8 19577696

